# Easy access to youth mental health services in the Netherlands

**DOI:** 10.1192/j.eurpsy.2024.45

**Published:** 2024-08-27

**Authors:** T. Van Amelsvoort

**Affiliations:** Psychiatry and Neuropsychology, Maastricht University, Maastricht, Netherlands

## Abstract

**Abstract:**

Mental health problems have increased following the pandemic and are associated with considerable health, economic and societal outcomes, particularly affecting youth. In co-creation with young people several European prevention and early intervention strategies to promote mental wellbeing of youth are currently being developed. The development and implementation of easy-access youth mental services across Europe will be presented and discussed. In addition pilot data of online, hybrid treatment platforms and self-management ecological momentary intervention apps will be presented. Ultimately the aim is: 1) to develop clinical guidelines, best practices, and policy recommendations to mitigate the youth mental health challenges and 2) improve (cost-) effectiveness of early intervention strategies for promotion and prevention in mental health, including enhancing mental health literacy, resilience and self-management, while 3) actively involving young people in the process of these innovative developments. To amplify the reach, campaigns designed in co-creation with young people, to increase awareness, literacy, wellbeing and help-seeking among young people, targeting schools, further-education colleges, universities and other specific settings will need to be developed, specifically paying attention to high-risk groups within this young population, including children of parents with mental disorders, migrants, young people growing up in poverty, those in/leaving care, and the LGBTQ+ community, with coordination across domains: schools, general practitioners, and specialized mental healthcare facilities.

**Disclosure of Interest:**

None Declared

